# Editorial: Emerging Pneumonia in Children

**DOI:** 10.3389/fped.2021.813034

**Published:** 2021-12-08

**Authors:** Hong-Ren Yu, Jong-Hau Hsu

**Affiliations:** ^1^Department of Pediatrics, Kaohsiung Chang Gung Memorial Hospital, Kaohsiung, Taiwan; ^2^Graduate Institute of Clinical Medical Sciences, College of Medicine, Chang Gung University, Taoyuan, Taiwan; ^3^Department of Pediatrics, Kaohsiung Medical University Hospital, Kaohsiung, Taiwan; ^4^Department of Pediatrics, School of Medicine, College of Medicine, Kaohsiung Medical University, Kaohsiung, Taiwan

**Keywords:** pneumonia, pediatric, pathogen, pathophysiology, treatment

Given the development and progress of antimicrobial therapy, effective vaccines for pneumonia, and related guidelines management in the past several decades, pneumonia persists as a leading cause of death in children younger than 5 years in developing countries and accounts for ~20% of childhood deaths. Although advanced imaging modalities including chest sonography, computed tomography, and magnetic resonance imaging are promising for more accurate and timely diagnosis, their roles in clinical management need further investigation. Even though there are various specific or sensitive laboratory tests for upper respiratory tract samples or sputum, which can reveal nucleic acid from potential pathogens in children, it is, unfortunately, difficult to attribute the cause due to limitations in our ability to distinguish between the colonized organisms or pathogens in the upper respiratory tract. In addition, the emergence of other novel pathogens such as Covid-19 presents further issues. Thus, better diagnostic and therapeutic strategies are essential for children, and diagnostic and therapeutic guidelines also need to be updated with new evidence.

Due to the importance of emerging pneumonia, this special volume of Frontiers in Pediatrics invited contributions that highlight recent developments in this field. The main aim of this Research Topic is to consider the available data on pathogenesis, pathogens, imaging modalities, treatment, and the prevention of pneumonia in children. This special volume comprises ten articles.

## Face Protection for Children in Healthcare Settings (Vlacha and Feketea)

The COVID-19 pandemic has had a huge impact on countries around the world. It is important to reduce the spread of the virus in medical institutions between patients and medical staff. The first study in this volume provides evidence from the real world, showing the effects of state government mandates for face mask use in public. More than 200,000 COVID-19 cases were estimated to be avoided. The findings suggest that requiring face mask use in public could help in mitigating the spread of COVID-19. The CDC recommends that pediatric patients receive enhanced barrier prevention measures including face masks after entering the outpatient setting. However, due to the special characteristics of young children, the implementation of safety measures in outpatient pediatric centers can sometimes be extremely challenging. Based on personal experience, Vlacha and Feketea recommend the use of face shields for children who refuse to wear masks. Young children seem to tolerate face shields better than masks. Face protection equipment can ensure social distance, avoid the risk of suffocation and eliminate children's facial touching behavior. More evidence is expected to support the beneficial effect of face shields for children.

## Higher Hospitalization Rate For Lower Airway Infection in Transfusion-Naïve Thalassemia Children (Vlacha and Feketea)

Thalassemia is common among people of Italian, Greek, Middle Eastern, South Asian, and African descent. In China, the prevalence of α-thalassemia, β-thalassemia, and α + β-thalassemia were estimated at 7.88, 2.21, and 0.48%, respectively. Non-transfusion-dependent thalassemia patients can be at risk of ineffective erythropoiesis, peripheral hemolysis, and iron overload that contributes to a number of clinical morbidities. However, few articles discuss the infection susceptibility of non-transfusion-dependent thalassemia patients. Tsai et al. conducted a nationwide population-based retrospective cohort study using the Taiwan National Health Insurance Research Database. After confounding factors were adjusted, the hospitalization rate and incidence rate of bronchitis/bronchiolitis and pneumonia for transfusion-naïve thalassemia children were found to be higher than those for non-thalassemia controls. However, the exact pathologic mechanism needs to be further clarified. Whether the transfusion-naïve thalassemia patients also have a higher risk of other types of infection, such as urinary tract infection and acute gastroenteritis is worth further study. This finding reminds us to pay more attention to lower airway infection of non-transfusion-dependent thalassemia children in clinics.

## Clinical Features and Temporal Changes of RT-PCR and Chest CT in COVID-19 Pediatric Patients (Wei et al.)

Viral nucleic acid testing and chest CT have been considered as the main diagnostic methods for patients with suspected COVID-19 pneumonia. In adults, the positive rate of pharyngeal swabs reverses transcription polymerase chain reaction (RT-PCR) and is sometimes lower than that of RT-PCR in certain conditions, and chest CT is considered a more accurate early diagnostic tool. In contrast to adults, children with COVID-19 infection often present mild manifestation. Besides, children are more sensitive to radiation exposure than adults. Thus, a reasonable diagnostic approach for children suspected to have COVID-19 infection should be different from adults. In this study, Wei et al. investigated the clinical features and changes of RT-PCR and chest CT in COVID-19 children. They found that lung involvement in children with COVID-19 is mild, even with 28% of them as normal in CT. They concluded RT-PCR is more reliable than chest CT in the initial diagnosis of pediatric patients with COVID-19. Research from Guo et al. reflects the limitation of chest CT in the diagnosis of COVID-19 children.

## Noninvasive Ventilation and Mechanical Insufflator-Exsufflator for Acute Respiratory Failure in Children With Neuromuscular Disorders (Chen and Hsu)

Non-invasive ventilation and secretion clearance strategy is a recent trend for patients of neuromuscular disease with respiratory failure. This strategy has the advantages of convenience, life-quality preservation, and low cost. In this review, Chen and Hsu. describe recent advances in this non-invasive strategy for this particularly vulnerable group of patients who are often threatened by respiratory failure or airway obstruction by inadequate clearance of secretions during respiratory infections.

## Features Discriminating COVID-19 From Community-Acquired Pneumonia in Pediatric Patients (Guo et al.)

The clinical symptoms of COVID-19 pneumonia in children are similar to other pediatric community-acquired pneumonia but they have a different treatment strategy. It is important to distinguish COVID-19 from other pediatric pneumonias. Guo et al. compared the clinical, laboratory, and radiological results of COVID-19 children collected during the COVID-19 outbreak with other pneumonia patients collected before the COVID-19 outbreak in the same hospital, especially mycoplasma pneumonia. They found that no reliable specific features could discriminate COVID-19 from community-acquired pneumonia in pediatric patients. Even though more COVID-19 cases showed ground-glass opacity on CT scan and elevated level of ALT than other community-acquired pneumonia. A reliable and specific RT-PCR or antigen screening is necessary for quickly and accurately identifying the pathogen.

## COVID-19 Pneumonia in Children: From Etiology to Management (Parisi et al.)

In this review article, Parisi et al. summarize the characteristics of COVID-19 in children, including pathological mechanisms, clinical manifestation, radiologic findings, and therapeutic strategies. For their unique immune response and ACE2 expression, the clinical manifestation of COVID-19 in pediatric patients is often much less severe than in adults, progression of the disease remains possible and should be intercepted with appropriate treatment. Besides children are important vectors of COVID-19. In this article, Parisi et al. also provided a clear graphic abstract illustrating the virus-host interaction and reasons why children are less affected.

## Post-Infectious Bronchiolitis Obliterans: HRCT, DECT, Pulmonary Scintigraphy Images, and Clinical Follow-Up in Eight Children (Chen et al.)

Bronchiolitis obliterans (BO) is an uncommon but challenging lung disease for pediatricians. It is characterized by chronic irreversible inflammation and limited treatment strategy, thus it is important to make a timely diagnosis and start treatment promptly. High-resolution CT (HRCT) is a widely used imaging modality for the diagnosis of BO, and scintigraphy is an alternative tool that can functionally evaluate BO. Another choice of imaging modality, dual-energy computed tomography (DECT), has also recently been applied to BO. In this retrospective study, Chen et al. showed that the most common HRCT finding of BO is a mosaic pattern, where matched ventilation/perfusion (V/Q) defect is an essential feature in pulmonary scintigraphy. Furthermore, they found that DECT could reveal various degrees of decreased perfusion, which was compatible with the decreased perfusion on pulmonary scintigraphy.

## COVID-19 In Children: Respiratory Involvement and Some Differences With the Adults (Hernández and Orozco)

In this review, Hernández and Orozco summarize some of the mechanisms and findings that are different between adult and pediatric COVID-19 infections. They also review some important issues about the physiopathology, diagnosis, clinical and paraclinical presentation, severity, treatment, and control of the disease. Immune responses, microbiota effects, ACE2 activity, and more preserved coagulation and endothelial function account for the clinical advantages that contribute to milder presentation in children with COVID-19 infection. CT scan is suggested if there is clinical worsening or suspicion of pulmonary embolism. Pediatric treatment focuses on supportive care as there is less research into vaccines and specific pharmacotherapy. There are still knowledge gaps in pediatric COVID-19 and further research on children is required.

## All You Need is Evidence: What We Know About Pneumonia in Children With Neuromuscular Diseases (Cherchi et al.)

Respiratory failure is the most common cause of mortality in patients with neuromuscular diseases (NMD). Respiratory weakness is defined as the inability of the respiratory muscles to generate sufficient levels of pressure and flow to overcome the respiratory load. Alveolar hypoventilation will develop first during sleep and then progress to involve wakefulness. In this review, Cherchi et al. summarize the pathophysiology, issues of inadequate clearance, respiratory interventions, and future therapeutic directions and shed some light on the more comprehensive understanding and management of pneumonia in children with NMD.

## Emergent Pneumonia in Children (Perret et al.)

After the outbreak of the COVID-19 pandemic, emerging pneumonia has become an important issue for pediatricians across all fields. In this review, Perret et al. classify emerging pathogens into three categories: 1) true emerging: SARS-CoV-1, SARS-CoV-2, avian influenza, MERS-CoV, and hantavirus; 2) re-emerging, including measles, tuberculosis, and antimicrobial resistant bacteria such as CA-MRSA, Pseudomonas aeruginosa, Acinetobacter baumannii, Stenotrophomonas maltophilia, Mycoplasma pneumoniae, and new serotypes of post-vaccine pneumococcal; and 3) old known with new presentations, including rhinovirus, and non-SARS coronavirus. In this article, the epidemiology, forms of presentation, therapy, and prognosis in children are comprehensively described and compared with those in adults.

## Author Contributions

H-RY and J-HH contributed to the writing of the manuscript. All authors contributed to the article and approved the submitted version.

## Funding

This article was supported in part by a grant CMRPG8K0071 (H-RY) from Chang Gung Memorial Hospital.

## Conflict of Interest

The authors declare that the research was conducted in the absence of any commercial or financial relationships that could be construed as a potential conflict of interest.

## Publisher's Note

All claims expressed in this article are solely those of the authors and do not necessarily represent those of their affiliated organizations, or those of the publisher, the editors and the reviewers. Any product that may be evaluated in this article, or claim that may be made by its manufacturer, is not guaranteed or endorsed by the publisher.

